# High-throughput low-cost nl-qPCR for enteropathogen detection: A proof-of-concept among hospitalized patients in Bangladesh

**DOI:** 10.1371/journal.pone.0257708

**Published:** 2021-10-01

**Authors:** Katelyn E. Flaherty, Jessica A. Grembi, Vasavi V. Ramachandran, Farhana Haque, Selina Khatun, Mahmudu Rahman, Stace Maples, Torben K. Becker, Alfred M. Spormann, Gary K. Schoolnik, Andrew J. Hryckowian, Eric J. Nelson

**Affiliations:** 1 Department Environmental and Global Health, University of Florida, Gainesville, Florida, United States of America; 2 Department of Emergency Medicine, University of Florida, Gainesville, Florida, United States of America; 3 Department of Civil and Environmental Engineering, Stanford University, Stanford, California, United States of America; 4 Department of Medicine, School of Medicine, Stanford University, Stanford, California, United States of America; 5 Department of Pediatrics, School of Medicine, Stanford University, Stanford, California, United States of America; 6 Institute of Epidemiology, Disease Control and Research, Ministry of Health and Family Welfare, Government of Bangladesh, Dhaka, Bangladesh; 7 Institute for Global Health, University College London, London, United Kingdom; 8 International Centre for Diarrhoeal Disease Research, Bangladesh, Dhaka, Bangladesh; 9 Geospatial Center, Branner Library, Stanford University, Stanford, California, United States of America; 10 Department of Medical Microbiology and Immunology, University of Wisconsin-Madison, Madison, Wisconsin, United States of America; 11 Department of Medicine, University of Wisconsin-Madison, Madison, Wisconsin, United States of America; 12 Department of Pediatrics, University of Florida, Gainesville, Florida, United States of America; CINVESTAV-IPN, MEXICO

## Abstract

**Background:**

Diarrheal disease is a leading cause of morbidity and mortality globally, especially in low- and middle-income countries. High-throughput and low-cost approaches to identify etiologic agents are needed to guide public health mitigation. Nanoliter-qPCR (nl-qPCR) is an attractive alternative to more expensive methods yet is nascent in application and without a proof-of-concept among hospitalized patients.

**Methods:**

A census-based study was conducted among diarrheal patients admitted at two government hospitals in rural Bangladesh during a diarrheal outbreak period. DNA was extracted from stool samples and assayed by nl-qPCR for common bacterial, protozoan, and helminth enteropathogens as the primary outcome.

**Results:**

A total of 961 patients were enrolled; stool samples were collected from 827 patients. Enteropathogens were detected in 69% of patient samples; More than one enteropathogen was detected in 32%. Enteropathogens most commonly detected were enteroaggregative *Escherichia coli* (26.0%), Shiga toxin-producing *E*.*coli* (18.3%), enterotoxigenic *E*. *coli* (15.5% heat stable toxin positive, 2.2% heat labile toxin positive), *Shigella* spp. (14.8%), and *Vibrio cholerae* (9.0%). Geospatial analysis revealed that the median number of pathogens per patient and the proportion of cases presenting with severe dehydration were greatest amongst patients residing closest to the study hospitals.”

**Conclusions:**

This study demonstrates a proof-of-concept for nl-qPCR as a high-throughput low-cost method for enteropathogen detection among hospitalized patients.

## Introduction

Approximately 4.5 billion people are impacted by diarrheal disease each year [1]. Advances in molecular diagnostics paired with case-control studies have granted a more granular understanding of the agents to which disease can be attributed (etiologic) versus detected agents to which disease cannot be attributed (non-etiologic). PCR-based diagnostics have attributed upwards of 65–89% of diarrheal cases to enteropathogens [2]. In a multi-center community case-control study among children under 2 years of age with diarrheal disease in resource limited settings (MAL-ED), the percentage of diarrheal cases attributed to viruses, bacteria, and parasites are 36%, 25%, and 4% respectively [3]. The five most common etiologic agents were *Shigella* spp., sapovirus, rotavirus, adenovirus, and enterotoxigenic *Escherichia coli* (ST-ETEC) [3]. In a multi-center case-control study among children under 5 years of age with diarrheal disease that sought clinical care in resource limited settings (GEMS), the five most common etiologic agents were *Shigella* spp. rotavirus, adenovirus, heat-stable enterotoxigenic *Escherichia coli* (ST-ETEC) and *Cryptosporidium* spp [2]. Co-infections are common and have been shown to have variable additive risk of severe dehydration [4, 5]. There is a high burden of carriage of enteropathogens in low-and-middle income countries. In Bangladesh, rates of enteropathogen detection from asymptomatic children under 5 is as high as 44% [6]. Less is known about the etiology and carriage of enteropathogens in adult populations [7–9].

Laboratory diagnostic approaches are often governed by location, epidemiology, and resources. Culture and PCR are gold standards for enteropathogen identification. Less sensitive immunologic rapid diagnostic tests are important in settings without conventional laboratory access [10–12]. In global health research, customized TaqMan Array Cards (TAC) are a robust and well-accepted quantitative PCR (qPCR) method with equivalent to better results than culture [13, 14]. Despite the strengths of TAC, it is often cost-prohibitive at 60 USD per sample; thus, TAC is largely limited to research with substantial funding [13]. One alternative was developed by Grembi et al. with the objective to decrease cost 6-fold while maintaining similar clinical diagnostic capability [15]. The technique uses nano-liter qPCR (nl-qPCR) on a 5184-well chip commercially available by the Takara Bio Group (Shiga, Japan; formerly developed by Wafergen, Biosystems, CA, USA). Cost savings compared to TAC are achieved by the high-throughput nature of the platform and less expensive reagents costs (e.g. SYBR Green). When analytically tested by spiking synthetic targets into stool, the sensitivity and specificity of the nl-qPCR assay ranged for 98%-100% and 90%-100% across targets, respectively [15]. The nl-qPCR approach was slightly less sensitive than TAC for some targets and nanofluidic volumes result in limits of detection of 1–2 orders of magnitude less than TAC. Despite these limitations, nl-qPCR offers an intriguing opportunity in global health to expand diarrheal disease research at an estimated cost of 8–12 USD per sample. This nl-qPCR method requires the capacity to ship nucleic acid extractions in 96-well plates to a centralized laboratory with the nl-qPCR instrument and a liquid handling robot; thus, the approach may also be restricted to research settings.

Initial validation and deployment of the nl-qPCR method was conducted with synthetic standards and stool samples to identify enteropathogens from asymptomatic non-hospitalized children (median age 14 +/- 2 months) in Bangladesh [15]. The etiologic cutoffs for most pathogens were within the sensitivity and limit of detection (LOD) of the assays. However, the study was limited by a lack of confirmation with symptomatic patients of all ages. To address this limitation, we conducted a two-step study to identify enteropathogens in stool samples collected from hospitalized children and adults suffering from diarrheal disease in Bangladesh. In the first step, we focused on *Vibrio cholerae* to evaluate nl-qPCR among hospitalized patients because the high caseload in the study area assured sufficient sample size to compare nl-qPCR against the gold standards of conventional qPCR and culture [10, 16, 17]. Using latent class modeling, culture had a sensitivity of 57.1% (95% CI 40.4–73.2) and specificity of 99.7 (99.3–99.9), and nl-qPCR had a superior sensitivity of 97.6% (95%, CI 89.0–100.0) and a similar specificity of 99.6% (98.3 to 100.0). In the second step herein, we demonstrate a proof-of-concept for the detection of a broad panel of bacterial, helminth, and protozoal enteropathogens by nl-qPCR among hospitalized patients seeking hospital care for diarrheal symptoms; several targets are difficult to detect by conventional techniques or less common making cross platform validations less tractable.

## Methods

### Ethics statement

The process for recruitment, enrollment and written informed consent/assent has been described previously [16]. Ethical approvals were obtained at the Institutional Review Boards (IRBs) of Stanford University School of Medicine and the Institute of Epidemiology, Disease Control and Research (IEDCR) within the Bangladesh Ministry of Health and Family Welfare. Written informed consent was obtained from the parent/guardian of each participant under 18 years of age.

### Study design

The study site, population, and enrollment have been described previously [16]. In brief, the study was conducted at two government hospitals in the rural district of Netrokona, Bangladesh: Netrokona District Hospital and Madan Upazila Health Complex. Netrokona was selected as the study location due to its remote location, resource limitations, and frequent outbreaks of diarrheal diseases [16, 18]. The population of Netrokona is approximately 2.2 million with approximately 5000 cases of diarrheal disease monthly [11]. Participants were enrolled from September 7^th^ to December 29^th^, 2015 when cholera outbreaks are common [4, 19]. Samples were collected as part of a clinical study in which digital clinical decision-support was found to improve the quality of dehydration assessment [16]. Digital decision-support was used from November 15^th^ to December 29^th^, 2015. The inclusion criteria were patients at least 2 months of age presenting with acute (<7 days) diarrhea (>3 loose stools in 24 hours) at the two hospitals. Patients with complications or co-morbidities were excluded; patients with severe malnutrition were included prior to integration of the Rehydration Calculator on November 15^th^, after which they were excluded due to clinical limitations.

### Laboratory procedures

#### Sample collection

Collection methods were described previously [17]. Two ml stool samples were collected and placed in 6 ml of Invitrogen RNAlater (75% final RNAlater concentration), stored on site at 4°C and later frozen at -80°C.

#### Molecular analysis

Procedures have been previously described [15]. In brief, DNA from stool samples in 75% RNAlater (Invitrogen) was extracted using the MoBio PowerSoil 96-well kit. Extracts were assayed using a 5184-well nl-qPCR chip with assays for virulence and marker genes of 17 bacterial, protozoal and helminth enteropathogens [15] (S1 Table). These agents were targeted because antimicrobial agents are indicated and/or the agents are common enteropathogens [20]. Roche LightCycler 480 SYBR Green master mix was used to amplify DNA. PCR conditions were 95°C for 3 minutes followed by 40 two-step cycles at 95°C for 60 seconds and 60°C for 70 seconds. Cycle threshold (Ct) values of equal to or less than an average of 28 were considered positive based on previously performed standard curves with synthetic templates that found a limit of detection of 10–100 copies per reaction (100 nl volume); GenBank accession numbers for the synthetic standards are available in the publication by Grembi et al. [15]. Controls were (i) *V*. *cholerae* E7946 at 1 x 10^8^ Colony Forming Unit per gram (CFU/g) and 5 x 10^8^ CFU/g in 75% RNALater as extraction controls per 96-well plate with the clinical samples [21], (ii) additional extraction controls in a dedicated control plate of enterotoxigenic *Escherichia coli* Vm75688 (ETEC; ST+/LT+; 1.3 x 10^8^ CFU/g, stdev 0.22 x 10^8^ CFU/g), and *E*. *coli* E344206 (ST-/LT-; 0.9 x 10^8^ CFU/g, stdev 0.075 x 10^8^ CFU/g) in 75% RNALater plus *V*. *cholerae* E7956 at 1x10^8^ CFU/g with and without a 375 mg spike of control human stool in 75% RNAlater in the same plate [22, 23], (iii) phocine herpesvirus synthetic DNA (PhHV; IDT gblock 490bp) added to the mastermix and then amplified with PhHV primers to monitor for performance across chips and detect PCR inhibitors [3], and (iv) two no template controls (NTCs) per plate with the clinical samples. All samples were run in two technical replicates.

### Data analysis

#### Molecular analysis

Samples did not meet molecular quality control criteria if nl-qPCR results returned a Ct value of 28 or greater for either the PhHV spike-in or 16S rRNA gene targets. Pathogen counts were enumerated using an algorithm that accounted for the possibility of multiple genetic targets for a given enteropathogen (S1 Table). Primary technical validations of the method were described previously [15]. Data are presented in copy number of the genetic target detected per gram of stool using the formula 10^((Ct- intercept)/slope)) / 0.0125μl reaction volume x 100μl elution volume / 375mg stool x 1000mg/1gm; slope and intercept were obtained from chips run concurrently with those from this project.

#### Statistical analysis

The MAL-ED case-control study of children under 2 years of age found that detection of Enteroaggregative *E*. *Coli* (EAEC), atypical Enteropathogen *E*. *Coli* (aEPEC), and *Giardia lamblia* could not be attributed to symptomatic disease [24]; thus these 3 enteropathogens were removed from calculations involving etiologic pathogens [24]. Analyses for correlates to severe-dehydration were narrowed to the data collected between November 15, 2015 to December 29, 2015 when digital decision-support (a.k.a. Rehydration Calculator) was used to limit confounding from potentially unreliable low quality dehydration assessments made without digital decision-support [16]. Coinfections were enumerated and grouped according to the number of pathogens (0, 1, 2, > = 3). Odds ratios, with a reference of no enteropathogen detected, were calculated to identify correlates to severe dehydration. A Fischer’s exact test was used to test the null hypothesis of no statistical difference in severe dehydration rates between cases where a pathogen was identified (single agent or co-infection) and cases in which no pathogen was identified (two-tailed; alpha <0.01). Analyses were performed with R version 4.0.3 and the epitools package [25, 26]. Figures were produced using GraphPad Prism 8.0.1 [27].

#### Geospatial analysis

Data were recorded electronically by means of software (Outbreak Responder version 0.9) developed for this study. The software is a data collection tool for public health and research professionals. It is built specifically for outbreaks in resource limited settings with limited connectivity. The median number of pathogens per patient per administrative unit (union) were mapped. The proportion of diarrheal cases presenting with severe dehydration after November 15^th^ per union were mapped. To address confounding from non-study hospitals that were closer to participant households, Thiessen polygons were generated to demarcate nearest government district and subdistrict hospitals using straight-line distances; this approach enabled analysis of the sub-set of participants whose closest government hospital was the Netrokona District Hospital or Madan Upazila Health Center. The Getis-Ord Gi* statistic was used to compare median number of pathogens per patient and proportion of diarrheal cases presenting with severe dehydration amongst unions. This method calculates a Z-score and corresponding p-value for each union. Geospatial analyses were conducted in ArcGIS 10.7.1; basemap attributions are watermarked on figures by ArcGIS per ESRI policy [28, 29].

## Results

### Patient enrollment

**Fig 1:**
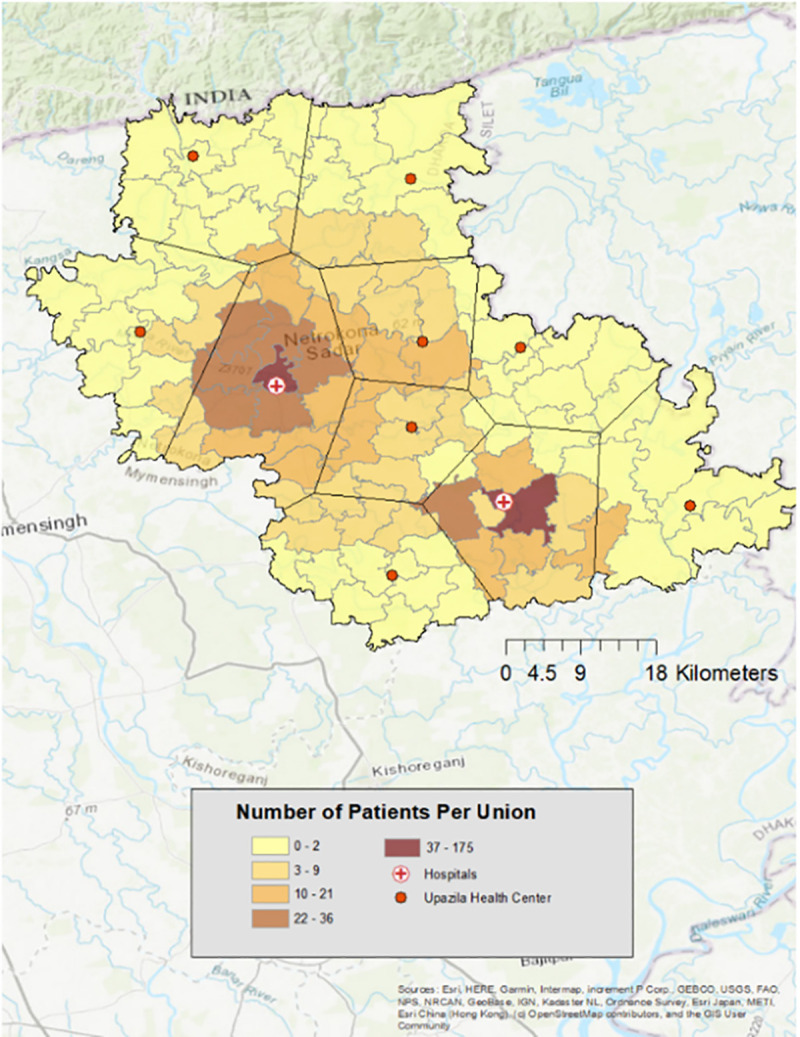
Geospatial distribution of 827 patients by administrative unit (union) and proximity to district hospitals. Basemap: World Topographic Map [29].

A total of 961 patients were enrolled at hospital admission (district hospital n = 723 district; subdistrict hospital n = 238); 123 of 426 (28.9%) and 82 of 511 (16.1%) were reported to have severe dehydration and were enrolled before and after Nov 15^th^, 2020, respectively (Table 1, Fig 1). A total of 110 patients did not have a stool sample collected/available. Patients (n = 17) reporting less than 3 loose stools in 24 hours (12 pre-Nov 15^th^, 5 post-Nov. 15^th^; 16 at district hospital, 1 at subdistrict hospital) were excluded, as well as those with samples that did not meet molecular quality control criteria (n = 7, 6 pre-Nov.15^th^,1 post-Nov.15^th^; 7 at district hospital). Analyses were conducted on the 827 patients that had stool samples collected (district hospital n = 602; subdistrict hospital n = 225).

**Table 1:** Patient characteristics.

Enrollment: n	827
Gender: n(%)	
Female	413 (49.9)
Male	414 (50.1)
Age: n(%)	
Under 5 years	373 (45.1)
5–17 years	61 (7.4)
18–44 years	230 (27.8)
45–64 years	132 (16.0)
65 years and older	31 (3.7)
Severe Dehydration: n(%)^a^	190 (23.0%)

^a^ As reported by the admitting physician (independent of digital decision support).

### Enteropathogen detection

**Fig 2:**
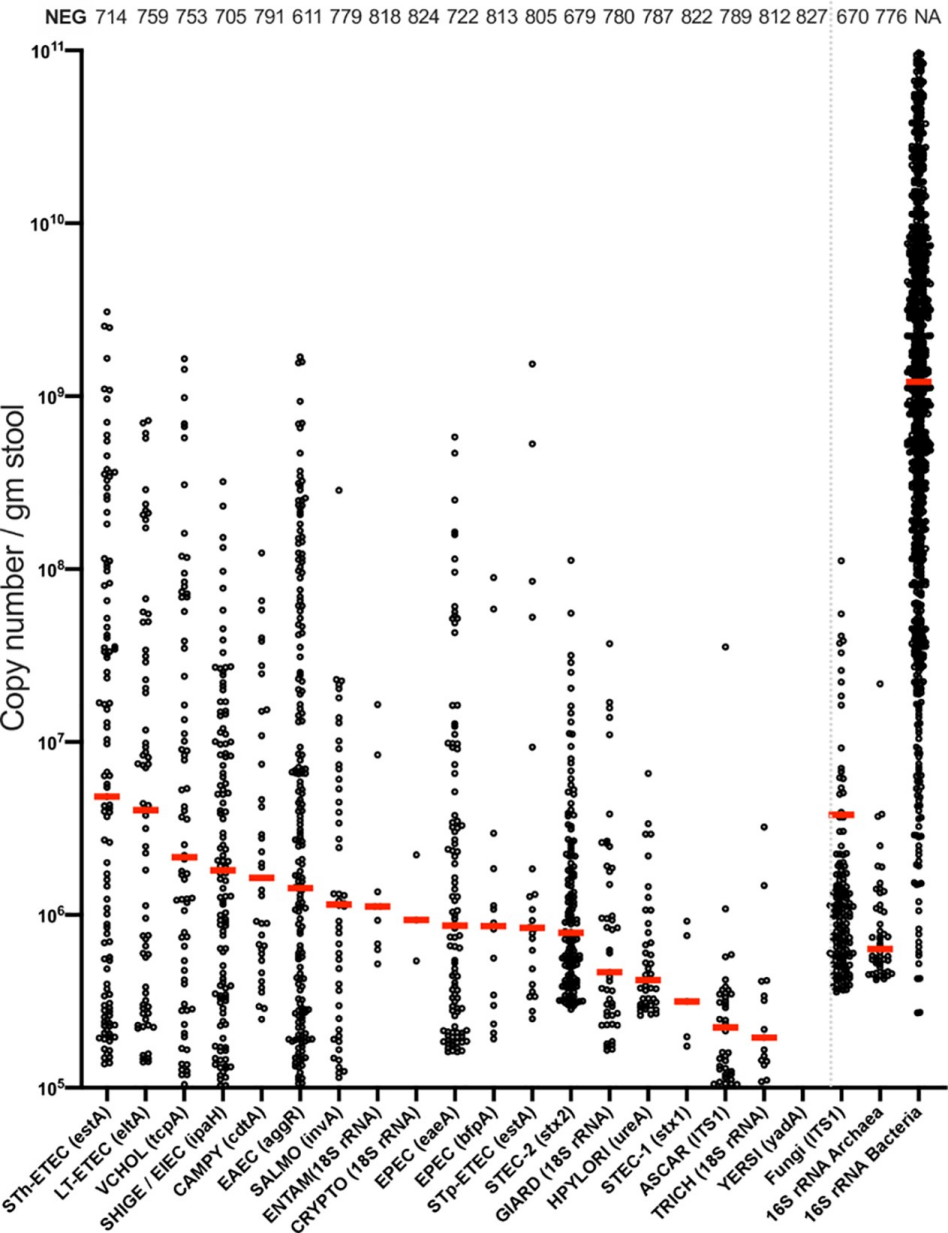
Enteropathogens detected by nl-qPCR. Red lines represent the median copy number per gram of stool. Vertical gray dotted line demarcates analysis of total Fungi, total Archaea, and Bacteria. Genetic targets are provided in brackets. Abbreviations are heat-labile and heat-stable enteropathogenic *Escherichia coli* (LT-ETEC and ST-ETEC), *Vibrio cholerae* (VCHOL), *Shigella* spp. (SHIGE), typical enteroaggregative *Escherichia coli* (EAEC), *Salmonella* spp. (SALMO), *Campylobacter jejuni/coli* (CAMPY), enteropathogenic *Escherichia coli* (EPEC), *Giardia lamblia* (GIARD), Shiga-toxin producing *Escherichia coli* (STEC), *Entamoeba histolytica* (ENTAM), *Ascaris lumbricoides* (ASCAR), *Cryptosporidium* supp (CRYPTO), *Trichuris trichiura* (TRICH), *Helicobacter pylori* (HPYLO), and *Yersinia enterocolitica* (YERSI); see Methods and S1 Table. NEG = number.

Positive controls of *V*. *cholerae* and ETEC were detected below the 28 threshold for positivity. The *V*. *cholerae* control did not change with the addition of control non-diarrheal stool: CT values for 1.1 x 10^3^ CFU/ml were 27.3 and 27.9 with and without control stool addition; 1.1 x 10^4^ CFU/ml were 26.9 and 26.7 with and without control stool addition; 1.1 x 10^6^ CFU/ml were 21.6 and 22.5 with and without control stool. The distribution of genetic target copy number per gram stool, a proxy for pathogen concentration, are shown for each genetic target ordered from high to low with total Fungi, Archaea and Bacteria demarcated. The counts for negative results are enumerated (Fig 2). Extraction controls added to each plate for *V*. *cholerae* at 1 x 10^8^ CFU/ml and 5 x 10^8^ CFU/ml yielded a median of 1.1 x 10^8^ copies/gm (n = 8) and 4.3 x 10^8^ copies/gm (n = 8) for the *tcpA* target, respectively.

**Fig 3:**
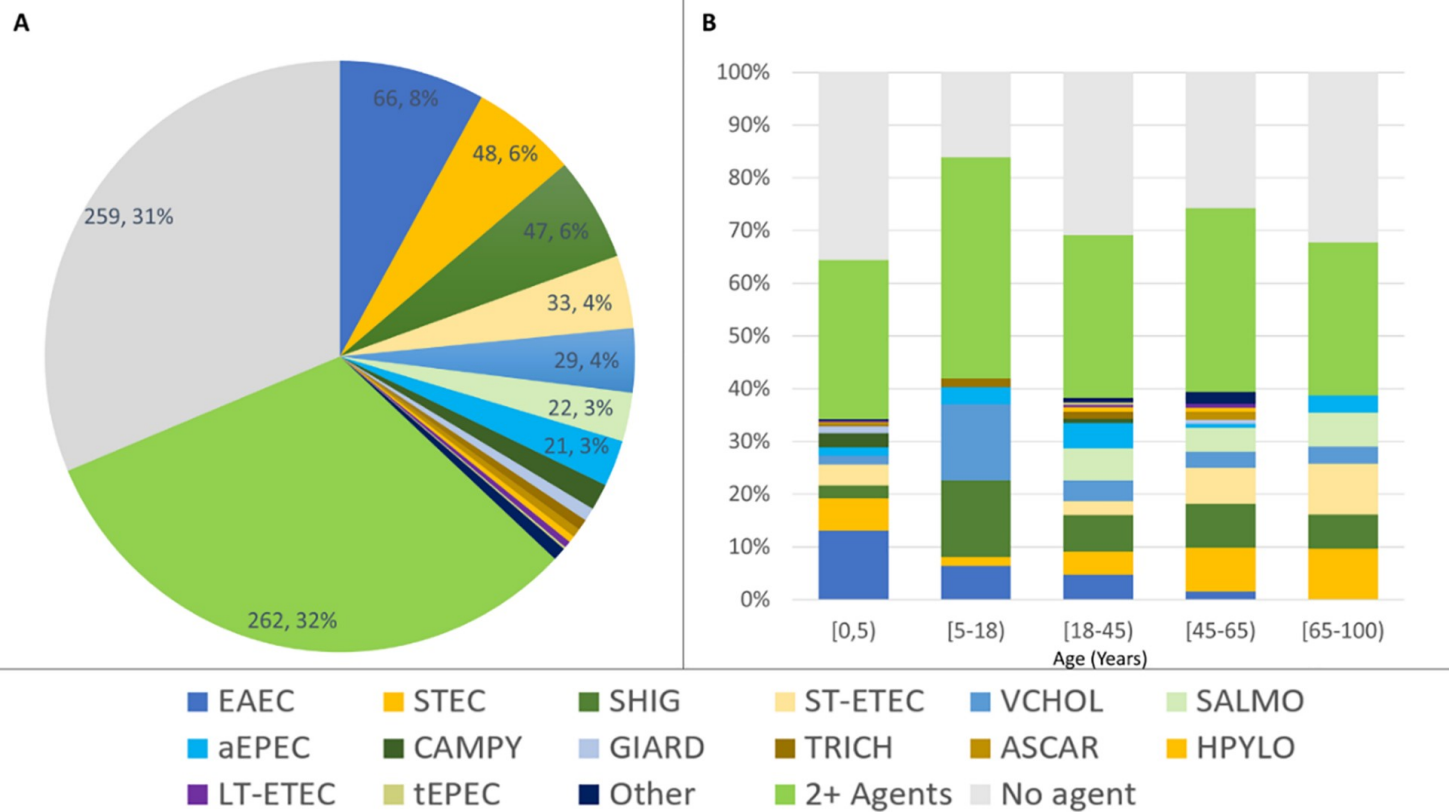
Distribution of single-and multi-pathogen infections among hospitalized patients with diarrheal disease. Distributions are presented as an aggregate **(A)** or stratified by age group **(B)**. The percentages are based on the total number of samples in which a stool sample was tested (n = 827). Pathogens under 1 percent are grouped as ‘other’. Abbreviations are defined in Fig 2.

**Fig 4:**
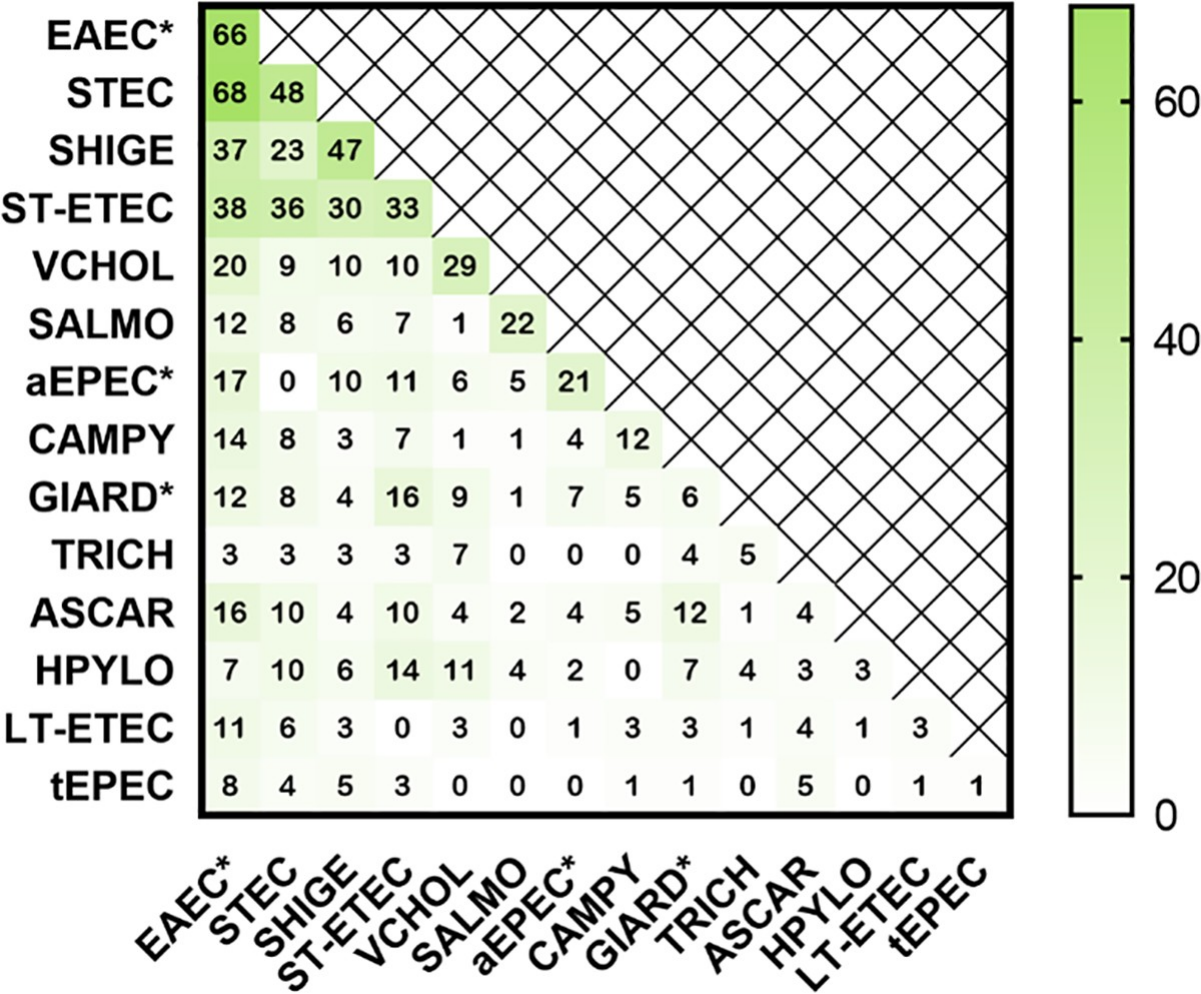
Distribution of co-infections. Data along the diagonal axis enumerate single pathogen detections. Shading represents the number of positive infections. Abbreviations are defined in Fig 2. Asterisks demarcate non-etiologic pathogens.

At least one pathogen was detected in 568 patients; 262 were infected with more than one pathogen (Fig 3). The most frequently identified enteropathogens were typical Enteroaggregative *E*. *coli* (EAEC, 26.0%, n = 215), Shiga-Toxin producing *E*. *coli* (STEC, 18.3%, n = 151), Heat-Stable Enterotoxigenic *E*. *coli* (ST-ETEC,15.5%, n = 128), *Shigella* spp. (14.8%, n = 122), and *V*. *cholerae* (9.0%, n = 74). The most commonly identified single enteropathogen infections were EAEC (66), STEC (48), *Shigella* spp. (47), ST-ETEC (33), and *V*. *cholerae* (29; Fig 3A). The most commonly identified single enteropathogen infections by age group were EAEC (<5 yrs), *Shigella spp*. and *V*. *cholerae* (5–17 yrs), *Shigella spp*. (18–44 yrs), *Shigella spp*. and STEC (45–65 yrs), and STEC and ST-ETEC (> = 65 yrs; Fig 3B). The most commonly identified coinfections included EAEC, a non-etiologic pathogen (Fig 4). Independent of EAEC detection, the most commonly identified coinfections involving 2 etiologic pathogens were ST-ETEC and STEC (36), ST-ETEC and *Shigella spp*. (30), and *Shigella spp*. and STEC (23; Fig 4).

#### Geospatial distributions for enteropathogens and severe dehydration

**Fig 5:**
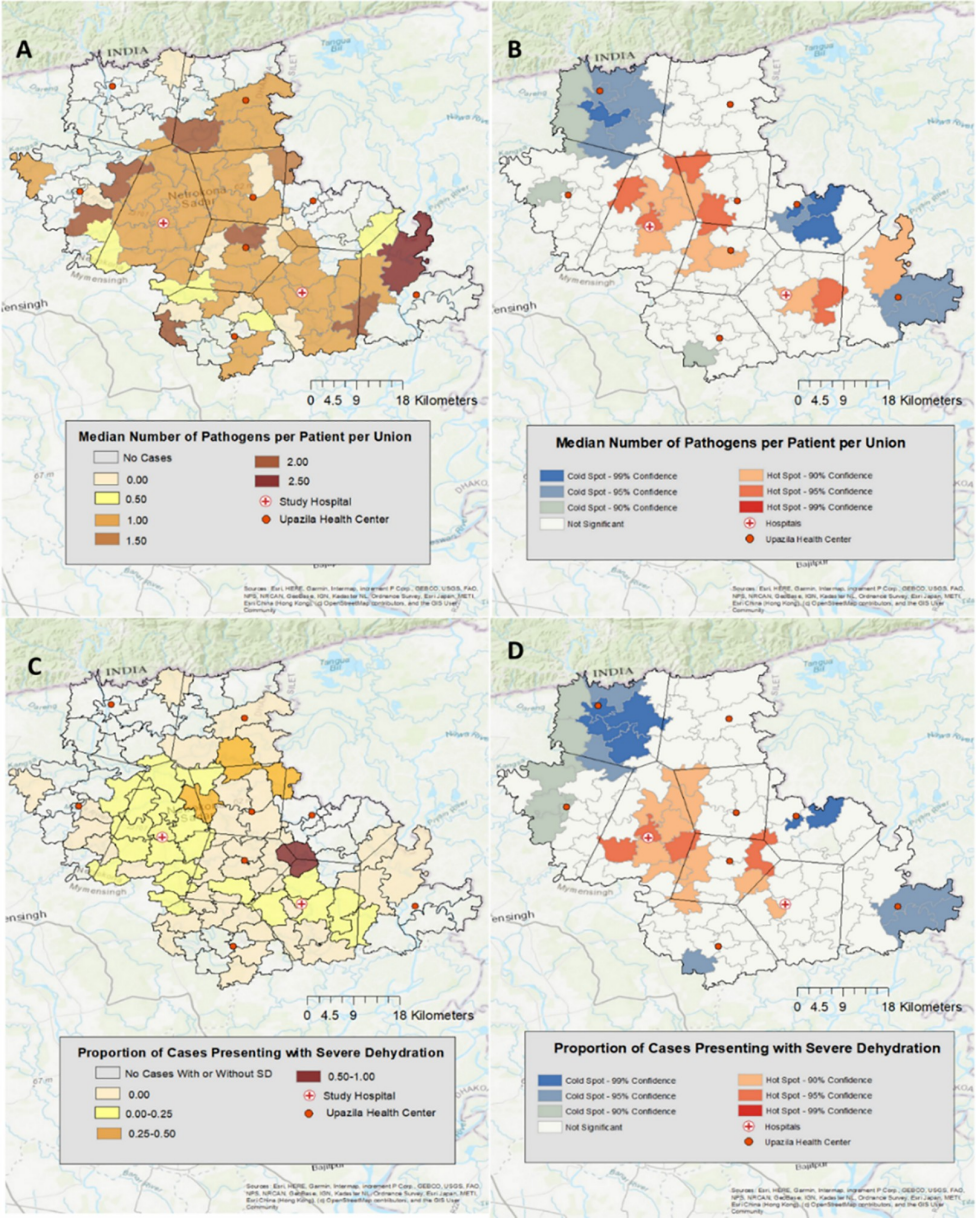
Geospatial distribution of diarrheal disease. **A.** Median number of pathogens per patient per administrative unit (union). **B.** Hotspot Analysis: Median number of pathogens per patient per union hot and cold spots. **C.** Proportion of cases presenting with severe dehydration per administrative unit (union) among subset of patients enrolled after November.15^th^. Data are derived from patients in which a stool sample was collected. **D.** Hotspot Analysis: Proportion of cases presenting with severe dehydration per union ‘hot’ and ‘cold’ spots. Basemap: World Topographic Map [29].

Geospatially, the median number of pathogens per patient per union was greater amongst patients residing in households closest to the 2 study hospitals (Fig 5A & 5B). Enteropathogen detections were evaluated as potential determinants of severe dehydration to identify high-risk infections requiring aggressive rehydration. There was no significant association between number of pathogens detected and severe dehydration (S2 Table). There was no significant difference in the odds ratio of severe dehydration between patients infected with *V*. *cholerae* compared to patients without an etiologic pathogen detected (OR = 1.43, CI: 0.48–3.70); similar findings were observed for the other enteropathogens (S3 Table). We analyzed the data for geospatial correlates of severe dehydration. The proportion of cases presenting with severe dehydration among households closest to Netrokona District Hospital (within the Thiessen polygon) was significantly greater than the proportion in patients residing outside of the Thiessen polygon for whom Netrokona District Hospital was not the closest hospital (Fig 5C). Among patients whose household was closest to Madan Upazila Health Complex (within the Thiessen polygon), the proportion of cases presenting with severe dehydration was not significantly greater than the proportion in patients residing outside of the Thiessen polygon surrounding Madan Upazila Hospital save for a single union (Fig 5D).

## Discussion

In this clinical diagnostic study of 827 patients presenting with acute diarrheal disease in Netrokona, Bangladesh who underwent systematic clinical and microbiologic investigation, we demonstrate a proof-of-concept for nl-qPCR as a high-throughput low-cost method for enteropathogen detection among symptomatic hospitalized patients.

We identified at least one pathogen in 69% of samples; most commonly EAEC, STEC, ST-ETEC, *Shigella* spp., and *V*. *cholerae*. These results are consistent with previous findings from a 22-year study among hospitalized patients in Dhaka, Bangladesh in which pathogens were identified in 62% of patients, most commonly *V*. *cholerae*, ETEC, and *Shigella* spp. using a mixture of molecular, immunologic, and culture techniques [4]. Our molecular results align with the molecular data (TAC) from both the MAL-ED [3] and GEMS [2] studies. While our study was not designed to calculate attributable ORs to define etiology for diarrheal symptoms, we found that ETEC (ST-ETEC) and Shigella were among the five most common pathogens detected. These enteropathogens were both characterized as etiologic for diarrheal disease and among the five most common agents in MAL-ED and GEMS. There were also findings that differed from MAL-ED and GEMs. For example, STEC (18.3%) was more common than Shigella (14.8%) and ST-ETEC (15.5%). To strengthen support for nl-qPCR and these clinical findings, future studies will benefit from the incorporation of viral targets in to the nl-qPCR platform and a comparative study of culture and TAC stool samples collected from hospitalized patients with diarrheal disease.

Among the subset of 511 patients who presented after the application of digital-decision support [16], we did not identify significant correlates to severe dehydration. The sample size of 82 severely dehydrated patients was insufficient to detect significant differences similar to those reported from the larger sample in a 22-year surveillance study conducted in Dhaka, Bangladesh [4]. Geospatially, we found that both the median number of pathogens and proportion of patients presenting with severe dehydration per union were greater in the urban areas closest to the study hospitals compared to the rural areas on the outskirts of Netrokona. These results are consistent with prior findings that suggest that urban settings have increased pathogen burden because of higher population density and risk of contamination between centralized water and sanitation systems [30]. As a result, this study indicates the need for continued research regarding the impacts of urban crowding and poor sanitation to better mitigate diarrheal disease [31, 32].

These findings must be viewed within the context of the study limitations. As this study was designed as a technical proof-of-concept for nl-qPCR, our secondary analyses for clinical correlates had insufficient sample size. This limitation was exacerbated by the subset analysis to the period after November 15^th^ when the Rehydration Calculator was deployed to assure dehydration assessments adhered to WHO guidelines. Furthermore, our analysis is limited to select non-viral enteropathogens. It is possible that patients without an identified enteropathogen were infected with a viral agent. This may have reduced the value of ‘no pathogen detected’ as a reference standard. In addition, we assumed data on etiology from patients under 5 years is relevant to symptomatic disease in patients older than 5 years because of a lack of case-control studies on etiology among patients older than 5 years. Moreover, the nl-qPCR methodology cannot yet differentiate hybrid *E*. *coli* pathotypes form coinfections [15]; thus, a proportion of the coinfections described herein may represent hybrid pathotypes. Procedurally, the plating efficiency of the extraction control was not evaluated by microscopy and therefore the precise extraction efficiency was not determined. With respect to external validity, the epidemiologic findings are limited to two study sites. Lastly, we were unable to determine attributable risk given the lack of non-diarrheal control samples.

## Conclusion

In this study, nl-qPCR was deployed to detect diarrheal pathogens among hospitalized patients in a low-and-middle income country as a proof-of-concept. The findings herein demonstrate that nl-qPCR can be used as an effective research tool to characterize enteropathogen burden. The approach taken herein can equip public health professionals with the evidence necessary to advance diarrheal disease mitigation and clinical guidelines.

## Supporting information

S1 Table Enteropathogen primers and probes. (PDF)

S2 Table Coinfection and severe dehydration from diarrheal disease. (PDF)

S3 Table Odds of severe dehydration by enteropathogen detected. (PDF)

S1 Dataset (CSV)
