# Supplementary material for: High-throughput low-cost nl-qPCR for enteropathogen detection: A proof-of-concept among hospitalized patients in Bangladesh
Source: PLoS One. 2021 Oct 1;16(10):e0257708. doi: 10.1371/journal.pone.0257708 (PMC8486112; doi:10.1371/journal.pone.0257708)
Supplement: S1 Table — (PDF) [file pone.0257708.s001.pdf]

S1 Table. Enteropathogen Primers and Probes

	Enteropathogen	Target	Sequence	Reference
Bacteria	*Campylobacter* spp.	*cdtA*	F:AAAGGATTTGGCGATGCTAGA<br>R:CCGCTGTATTGCTCATAGGG	Grembi et al. (1)
	EAEC	*aggR*	F:CAGCGATACATTAAGACGCCT<br>R:TCCTTTTGACCAATTCGGACA	Grembi et al. (1)
	EPEC [a]	*bfpA*	F:GCGAAAGGCTACGGTGTTAA<br>R:GCCTCAGCAGGAGTAATAGC	Grembi et al. (1)
		eaeA	F:GGTCAGATTCAGCATAGCGG<br>R:CGCGAGCGGTCACTTTATAA	
	ETEC [b]	*STh*	F:TTCACCTTTCGCTCAGGATG<br>R:CCCGGTACAAGCAGGATTAC	Grembi et al. (1)
		STp	F:ACTGAATCACTTGACTCTTCAAAAG<br>R:ACAACAAAGTTCACAGCAGTAAA	
		eltA	F:CCTGGATTCATCATGCACCA<br>R:TCTGGGTCTCCTCATTACAAGT	
	STEC [c]	*stx1*	F:ACAGATGGAATCTTCAGTCTCTTC<br>R:CTGAATCCCCCTCCATTATGAC	Grembi et al. (1)
		stx2	F:CTGTTAATGCAATGGCGGC<br>R:TGCAGTAACGGTTGCAGATT	
	H. pylori	*ureA*	F:AACTCGTAACCGTGCATACC<br>R:TGCCTTCGTTGATAGTGATGT	Grembi et al. (1)
	Salmonella spp.	*invA*	F:TTGACGGTGCGATGAAGTTT<br>R:CCACCGAAATACCGCCAATA	Grembi et al. (1)
	Shigella spp.	*ipaH*	F:GTCAGAAGCCGTGAAGAGAA<br>R:TTCAGTACAGCATGCCATGG	Grembi et al. (1)
	V. cholerae	*tcpA* [d]	F:ACACGATAAGAAAACCGGTCA<br>R:GCCTTGGTCATATTCTGCGA	Grembi et al. (1)
	Y. enterocolitica	*yadA*	F:GCCCAGAAAGATGGAGTAGC<br>R:CGTGACTAGAGTGTCCAATGG	Grembi et al. (1)
Protozoa	*Cryptosporidium* spp.	18S rRNA	F:GGGTTGTATTTATTAGATAAAGAACCA<br>R:AGGCCAATACCCTACCGTCT	Grembi et al. (1)
	E. histolytica	18S rRNA	F:ATTGTCGTGGCATCCTAACTCA<br>R:GCGGACGGCTCATTATAACA	Grembi et al. (1)
	Giardia lamblia	18S rRNA	F:GACGGCTCAGGACAACGGTT<br>R: TTGCCAGCGGTGTCCG	Grembi et al. (1)
Helminths	*Ascaris lumbricoides*	ITS1	F:GTAATAGCAGTCGGCGGTTTCTT<br>R:GCCCAACATGCCACCTATTC	Grembi et al. (1)
	Trichuris trichiura	18S rRNA	F:TTGAAACGACTTGCTCATCAACTT<br>R:CTGATTCTCCGTTAACCGTTGTC	Grembi et al. (1)
Total bacteria	NA	16S rRNA	F:GTGSTGCAYGGYTGTCGTCA<br>R:ACGTCRTCCMCACCTTCCTC	Grembi et al. (1)
Total archaea	NA	16S rRNA	F:ATTAGATACCCSBGTAGTCC<br>R:GCCATGCACCWCCTCT	Grembi et al. (1)
Total fungi	NA	ITS1	F:CTTGGTCATTTAGAGGAAGTAA<br>R:GCTGCGTTCTTCATCGATGC	Grembi et al. (1)

[a] typical EPEC positive is defined as detection of both *bfpA* and *eaeA;* atypical EPEC positive is defined as detection of *eaeA* without *bfpA*, *stx1* or *stx2*(1).

[b] ST-ETEC positive is defined as detection of STp or STh regardless if *eltA* is also detected, LT-ETEC positive is defined as detection of *eltA* without STp or STh (1, 2).

[c] STEC positive is defined as detection of either *stx1* or *stx2* (1). Only 60% of STEC strains contain the eaeA gene so we did not require an organism to contain eaeA to be called STEC (3, 4).

[d] *tcpA* primer has previously been designated as *tcpA*^{set2} by Nelson et al. (5)

Supplement References

1. Grembi JA, Mayer-Blackwell K, Luby SP, Spormann AM. High-Throughput Multiparallel Enteropathogen Detection via Nano-Liter qPCR. Front Cell Infect Microbiol. 2020;10:351.
2. Platts-Mills JA, Babji S, Bodhidatta L, Gratz J, Haque R, Havt A, et al. Pathogen-specific burdens of community diarrhoea in developing countries: a multisite birth cohort study (MAL-ED). Lancet Glob Health. 2015;3(9):e564-75.
3. De Rauw K, Jacobs S, Piérard D. Twenty-seven years of screening for Shiga toxin-producing Escherichia coli in a university hospital. Brussels, Belgium, 1987-2014. PLoS One. 2018;13(7):e0199968.
4. Karama M, Cenci-Goga BT, Malahlela M, Smith AM, Keddy KH, El-Ashram S, et al. Virulence Characteristics and Antimicrobial Resistance Profiles of Shiga Toxin-Producing Escherichia coli Isolates from Humans in South Africa: 2006-2013. Toxins (Basel). 2019;11(7).
5. Alexandrova L, Haque F, Rodriguez P, Marrazzo AC, Grembi JA, Ramachandran V, et al. Identification of Widespread Antibiotic Exposure in Patients With Cholera Correlates With Clinically Relevant Microbiota Changes. J Infect Dis. 2019;220(10):1655-66.
