# Supplementary material for: High-throughput low-cost nl-qPCR for enteropathogen detection: A proof-of-concept among hospitalized patients in Bangladesh
Source: PLoS One. 2021 Oct 1;16(10):e0257708. doi: 10.1371/journal.pone.0257708 (PMC8486112; doi:10.1371/journal.pone.0257708)
Supplement: S2 Table — (PDF) [file pone.0257708.s002.pdf]

**S2 Table.** Coinfection and severe dehydration from diarrheal disease

| Pathogens detected (all) | Total: n(%) | Subset n(%)[a] | Severe Dehydration [a] n(% of subset) | Odds Ratio | p-value[b] |
|---|---|---|---|---|---|
| 0 | 259(31.3) | 132(30.1) | 21(15.9) | ref | ref |
| 1 | 306(37.0) | 160(36.5) | 32(20.0) | 1.32(0.72-2.45) | 0.45 |
| 2 | 140(16.9) | 73(16.6) | 10(13.7) | 0.85(0.36-1.88) | 0.84 |
| >=3 | 122(14.8) | 74(16.9) | 12(16.2) | 1.03(0.46-2.21) | 1.00 |

| Etiologic pathogens detected[c] | Total: n(%) | Subset n(%)[a] | Severe Dehydration [a] n(% of subset) | Odds Ratio | p-value[b] |
|---|---|---|---|---|---|
| 0 | 334(40.4) | 173(39.4) | 28(16.2) | ref | ref |
| 1 | 321(38.8) | 169(38.5) | 33(19.5) | 1.25(0.72-2.20) | 0.48 |
| 2 | 109(13.2) | 58(13.2) | 8(13.8) | 0.84(0.34-1.90) | 0.84 |
| >=3 | 63(7.6) | 39(8.9) | 6(15.4) | 0.96(0.33-2.39) | 1.00 |

[a] Among patients enrolled after Nov 15th, 2020 when digital decision support was used.
[b] Fisher's exact test.
[c] Giardia, aEPEC, and EAEC were considered non-etiologic and removed from these analyses(1)

## Supplement References

1. Platts-Mills JA, Babji S, Bodhidatta L, Gratz J, Haque R, Havt A, et al. Pathogen-specific burdens of community diarrhoea in developing countries: a multisite birth cohort study (MAL-ED). Lancet Glob Health. 2015;3(9):e564-75.
