# Supplementary material for: High-throughput low-cost nl-qPCR for enteropathogen detection: A proof-of-concept among hospitalized patients in Bangladesh
Source: PLoS One. 2021 Oct 1;16(10):e0257708. doi: 10.1371/journal.pone.0257708 (PMC8486112; doi:10.1371/journal.pone.0257708)
Supplement: S3 Table — (PDF) [file pone.0257708.s003.pdf]

S3 Table. Odds of severe dehydration by enteropathogen detected

Pathogens Detected[a]: n					
	STEC	SHIGE	STETEC	VCHOL	SALMO
STEC	95				
SHIGE	21	74			
ST-ETEC	21	16	70		
VCHOL	4	7	6	28	
SALMO	6	1	2	0	16
Patients with Severe Dehydration [a]: n (%)					
	STEC	SHIGE	STETEC	VCHOL	SALMO
STEC	14 (14.7)				
SHIGE	4 (19.1)	11(14.9)			
ST-ETEC	3 (14.3)	2 (12.5)	10 (14.3)		
VCHOL	1 (25.0)	1 (14.3)	3 (50.0)	6 (21.4)	
SALMO	1(16.7)	0(0.0)	0(0.0)	0(0.0)	4(25.0)
Odds Ratios of Severe Dehydration [b]: OR (95%CI)					
	STEC	SHIGE	STETEC	VCHOL	SALMO
STEC	0.90(0.44-1.79)				
SHIGE	1.18(0.32-3.34)	0.91(0.41-1.91)			
ST-ETEC	0.84(0.18-2.57)	0.72(0.10-2.72)	0.87(0.38-1.86)		
VCHOL	1.77(0.06-15.44)	0.90(0.03-5.57)	4.98(0.84-29.53)	1.43(0.48-3.70)	
SALMO	1.08(0.04-7.13)	NA	NA	NA	1.78(0.45-5.56)

[a] Among patients enrolled after Nov 15th, 2020 when digital decision support was used.

[b] Odds ratio computes the single or co-infection data against the reference of samples in which no etiologic pathogen was detected.
